# Underlying determinants of health provider choice in urban slums: results from a discrete choice experiment in Ahmedabad, India

**DOI:** 10.1186/s12913-018-3264-x

**Published:** 2018-06-19

**Authors:** Vilius Černauskas, Federica Angeli, Anand Kumar Jaiswal, Milena Pavlova

**Affiliations:** 1 Department of Health Services Research, Care and Public Health Research Institute, Faculty of Health Medicine and Life Sciences, Maastricht University, Duboisdomein 30, P.O. Box 6200 MD, Maastricht, the Netherlands; 2 Department of Organization Studies, School of Social and Behavioural Sciences, Tilburg University, P.O. Box 90153, Warandelaan 2, Tilburg, 5000 LE The Netherlands; 3 Indian Institute of Management Ahmedabad, Vastrapur, Ahmedabad 380015 India

**Keywords:** Health provider choice, Urban slums, Health-seeking behaviour, Discrete choice experiment, Bottom of the pyramid

## Abstract

**Background:**

Severe underutilization of healthcare facilities and lack of timely, affordable and effective access to healthcare services in resource-constrained, bottom of pyramid (BoP) settings are well-known issues, which foster a negative cycle of poor health outcomes, catastrophic health expenditures and poverty. Understanding BoP patients’ healthcare choices is vital to inform policymakers’ effective resource allocation and improve population health and livelihood in these areas. This paper examines the factors affecting the choice of health care provider in low-income settings, specifically the urban slums in India.

**Method:**

A discrete choice experiment was carried out to elicit stated preferences of BoP populations. A total of 100 respondents were sampled using a multi-stage systemic random sampling of urban slums. Attributes were selected based on previous studies in developing countries, findings of a previous exploratory study in the study setting and qualitative interviews. Provider type and cost, distance to the facility, attitude of doctor and staff, appropriateness of care and familiarity with doctor were the attributes included in the study. A random effects logit regression was used to perform the analysis. Interaction effects were included to control for individual characteristics.

**Results:**

The relatively most valued attribute is appropriateness of care (β=3.4213, *p* = 0.00), followed by familiarity with the doctor (β=2.8497, p = 0.00) and attitude of the doctor and staff towards the patient (β=1.8132, *p* = 0.00). As expected, respondents prefer shorter distance (β= − 0.0722, *p* = 0.00) but the relatively low importance of the attribute distance to the facility indicate that respondents are willing to travel longer if any of the other statistically significant attributes are present. Also, significant socioeconomic differences in preferences were observed, especially with regard to the type of provider.

**Conclusion:**

The analyses did not reveal universal preferences for a provider type, but overall the traditional provider type is not well accepted. It also became evident that respondents valued appropriateness of care above other attributes. Despite the study limitations, the results have broader policy implications in the context of Indian government’s attempts to reduce high healthcare out-of-pocket expenditures and provide universal health coverage for its population. The government’s attempt to emphasize the focus on traditional providers should be carefully reconsidered.

## Background

The tremendous size of the population in India and the limited health care resources poses a challenge to sustain an adequate health care system. This system is full of paradoxes. India is both a destination for medical tourists seeking high-quality low-cost care [1] and at the same time, a place to live where access to public health care services is limited and leads to relatively high out-of-pocket expenses for local population [2]. Around 80% of outpatient care and 60% of inpatient care is provided by the private sector, which fails to provide affordable care for rural and poor urban populations [1]. Access to public facilities is not the only barrier. Nearly 60% of households indicate that poor quality of care is the main reason for not utilizing public health care facilities [3]. To illustrate further, the private expenditure on health made up around 70% of the total health expenditures in India in 2014 [4]. That is considerably higher than in many developed and developing countries. BoP populations, who live at the lowest income stratum of society and below the poverty line, are especially vulnerable to the lack of access to a timely, affordable and productive health care, because the cost of medical bills significantly contributes to the risk of catastrophic out-of-pocket expenditures [5]. This underscores the need for understanding what healthcare choices are made in the BoP setting along with their underlying determinants. Prior research further highlights the importance of health-seeking behaviour studies to produce knowledge in this sense [5, 6].

In addition to the above-mentioned challenges, India shows increasing morbidity from non-communicable diseases compared to that from communicable diseases, which is in line with the epidemiological transition [7]. The changing disease landscape shapes the demand for health care services. Comparable to most developing countries, medical pluralism, i.e. the presence of a variety of medical systems available to a given group [8], remains an important feature of the Indian health care system [9]. It is believed that traditional medicine is an answer to improving accessibility and affordability of care for BoP populations affected by non-communicable and lifestyle-induced health issues [9]. Correspondingly, Narendra Modi, the current prime minister of India, established the Ministry of Ayurveda, Yoga, Unani, Siddha, and Homeopathy (AYUSH) in 2014 to promote the use of traditional medicine as mainstream [9]. Little is known however regarding how various provider types are perceived and sought for by the population, particularly in the lowest income strata of society.

The aim of this paper is to examine the factors affecting the choice of health care provider in a low-income setting, specifically the urban slums in India. The existing literature on the reasons behind the choice of provider in developing countries is limited and primarily focussed on selected population or disease groups. Several researchers have explained health-seeking behaviour through socioeconomic factors as in studies conducted in Vietnam [10], in peri-urban Nepal [11], and in rural Ethiopia [12]. At the same time, studies have shown that service characteristics are salient determinants of choice of provider, but these studies focused on a specific disorder or population group as in the study performed among self-help group households in rural India [13], study on patients with schistosomiasis-related symptoms in Ghana [14], study on patients with chronic diseases in Malawi [15], study on rural women in India [16]. The highest concentration of studies exists on women and infant health-seeking behaviour within developing countries [17–24]. Therefore, there is a knowledge gap in understanding such behaviour patterns among the general population of urban slums in India.

Although the literature on determinants of provider choice is limited, most of the mentioned studies analyse the decisions based on actual choices post factum. These studies commonly use revealed-preference methods or data collected as part of larger household surveys and analyse which service or patient characteristics might explain the choice of provider and health-seeking behaviour in more general terms [25]. Only few studies made use of stated preference data collected employing discrete choice experiment (DCE) method to understand the salient factors behind the choice of provider. For example, Neuman, Neuman, and Neuman [26] explored the effect of experience on preferences for health care services in Israel; Hanson, McPake, Nakamba, and Archard [27] investigated the preferences for hospital quality in Zambia; Tang et al. [25] studied the preferences for public or private healthcare provision  among urban residents in China; Kruk, Rockers, Tornorlah Varpilah, and Macauley [28] explored population preferences for health care in Liberia; and Berhane and Enquselassie [29] looked at patients’ preferences for attributes related to health care services at hospitals in Ethiopia. India still remains an unchartered context, despite its unique health environment and the strong relevance of understanding the determinants of providers’ choice in this domain. India is characterized by better healthcare infrastructure than most developing countries, and by the strong presence of the traditional AYUSH healthcare system. Medical pluralism provides a particularly compelling case for understanding health-related stated preferences using DCE, which would enable health policy planners to achieve more effective care that also better matches individuals’ expectations [30].

## Methods

### Study setting and population

The research was conducted in Ahmedabad, capital of Gujarat, India. Close to India’s average, 72.5% of Gujarat’s population do not use public health facilities [3]. Ahmedabad is the largest city in the state and is also one of the fastest growing capitals in the world. Per latest available data, city’s population was around 6 million people. There are almost 1 million slum-dwellers in 229 thousand households living in 739 notified slums spread across all city zones [31]. The Ahmedabad’s slums served as the study’s setting.

### Study design

This study employed a DCE methodology. DCE is a quantitative method that assesses the strength of preferences and trade-offs influencing a choice [32], in our study, this is the choice of health care provider. The DCE form of stated preference study has been chosen as it is comparatively simple and best depicts the kinds of choices that individuals make in real life. DCE requires from respondents to make trade-offs among desired attributes and to indicate a preferred profile [33]. DCE assumes that while making a choice between two profiles (product or service characterized by a number of attributes) individuals derive utility from the attributes, rather than the product or service in itself, revealing stated-preferences through their choices [34]. The method is built upon assumptions of utility maximization and rationality, and has its theoretical background in the economic theory of random utility [33]. The following stages are common to DCE and were used in this study: (I) establishing attributes and meaningful levels for each attribute; (II) generating questionnaire through building of choice sets using experimental design; and (III) survey application and analysis [33].

The DCE method was first applied in studies in high-income countries. However, the applications of the method to low-income settings have been increasing [33]. As compared to other quantitative techniques of enquiry such as surveys, DCE questionnaires are relatively simple to administer, elicit intuitive understanding in the respondents, and therefore are particularly suited to resource-constrained settings with low literacy levels. Moreover, the multi-attribute nature of the presented scenarios is in line with the increasing importance of a multi-criteria, multi-dimensional holistic approach to the understanding of resource allocation and health priorities [35]. Prior literature provides clear guidelines related to the design of DCEs for low-income settings [33]. For instance, the choice of DCE attributes and attribute levels should be based on primary data, not solely on evidence from the literature. In addition, the cultural and language differences should be taken into account by pre-testing the research instrument. We took such measures when designing our DCE as we explain below.

#### Establishing attributes and levels

Previous research suggests to establish attributes and levels relevant to the study setting qualitatively [36]. In our study, this was done in two stages. First, the broad theoretical concept from general health-seeking behaviour studies [37, 38] on determinants influencing the choice of provider was used to understand the broad boundaries for relevant attributes. This was followed by a review of DCE studies conducted in developing countries that investigated health care utilization and a review of exploratory research that involved qualitative interviews on the matching topic in the same study setting [39]. As a result, a first extensive list containing potential attributes was formed. During the second step, several individual face-to-face interviews with both representatives of study sample and experienced local healthcare researchers were conducted to rank the potential attributes by importance to the local setting and establish the meaningful levels for these attributes. Specifically, respondents were given a list of pre-defined attributes and were asked to rank the attributes according to their relevance for the local setting in order to find the most relevant attributes. Respondents were also given an opportunity to name and discuss new attributes which were absent in the list of pre-defined attributes. We choose the attributes that were most frequently indicated as relevant by the respondents. The attributes indicated by interviewees as most relevant, were quite consistent. Thus, the analysis of interviews revealed five attributes that were most relevant and these were chosen to be included in the questionnaire. Similar approaches have been used in previous studies employing DCE e.g. [40].

The five selected attributes were: provider type and cost (through interviews it was found that price is closely linked with the type of provider, therefore, price was not included as a separate attribute and was rather used as a description of the provider type); distance to the facility; attitude of doctor and staff towards the patient; appropriateness of care; familiarity with the doctor. Four of these attributes were dichotomous, while provider type-and-cost attribute had three levels that included private allopathic provider, public allopathic provider and traditional (AYUSH) provider. Quantitative attribute levels were chosen for distance to the facility, while other attributes were given using qualitative levels. In addition to the attributes and their levels, a hypothetical situation (scenario) was developed to be portrayed to the respondents before filling the questionnaire. The developed scenario suggested the respondent to imagine that he/she needed to decide which health care facility to visit for a regular check-up in a case of chronic disease. Chronic disease was chosen due to its emerging prevalence in developing countries as discussed in the introduction. Table 1 shows the attributes, their levels and the coding chosen.

**Table 1:** Attributes and levels included in the study

Attribute	Attribute Levels and Regression Coding
Provider type and cost	Allopathic government facility – Rs. 100 per visit = 0
	Allopathic private facility – Rs. 300 per visit = 1
	Traditional provider (AYUSH) – Rs. 100 per visit = 1*
Distance to the facility	15 min = 15
	35 min = 35
Attitude of doctor and staff towards the patient	Friendly = 1
	Indifferent = 0
Appropriateness of care	The services provided are in line with the health requirements and personal circumstances = 1
	The services provided does not conform to the health requirements and personal circumstances = 0
Familiarity with the doctor	Known doctor = 1
	Unknown doctor = 0

In addition to the provider’s attributes, questions about socioeconomic characteristics of respondents were included, to take into account their potential influence on the hypothetical choice of provider. In line with previous work, questions on age, gender, marital status, education, household income and household size were included. Table 2 provides an overview of the individual socioeconomic characteristics and relative coding chosen.

**Table 2:** Coding of socioeconomic variables for the model with interactions

	Group	Coding
Age	18–39	0
	40–65	1
Gender	Male	0
	Female	1
Marital Status	Married	0
	Not married	1
Education	No formal education	0
	Formally educated	1
Household income per month	Low (Rs. 0–5000)	0
	Higher (Rs. 5000–25,000)	1
Household size	Small (1–4 members)	0
	Big (5–15 members)	1

#### Generating the questionnaire

The five attributes and their levels depicted in Table 1, give rise to a total of 48 (3^1^ × 2^4^) possible profiles (full factorial design). Therefore, a subset of profiles (fractional factorial design) was used to reduce the number of choice sets to reasonable number apprehensible to respondents. Specifically, orthogonal main effects design was generated using SPSS version 23 software producing a total of 8 scenarios. The assessment of the automatically generated profiles showed that the profiles set meets the necessary criteria for orthogonality, overlap and maximized level balance. Perfect level balance could not be achieved because one of the attributes has three levels.

One of the 8 profiles was chosen as a base profile and was matched against the other 7 profiles to form 7 choice tasks (see Table 3 for an example choice task). The first choice task was repeated in the questionnaire as a last choice item, leading to a total of 8 choice tasks. This was performed to check for validity, and establish whether respondents understood the task and made consistent choices. As a result, the questionnaire contained 8 choice tasks, each containing the base (fixed) profile and one alternative profile from the orthogonal design.

**Table 3:** Example choice task

	Profile A	Profile B
Provider type and cost	Allopathic government facility – Rs. 100	Allopathic government facility – Rs. 100
Distance to the facility	35 min	35 min
Attitude of doctor and staff towards the patient	Indifferent	Indifferent
Appropriateness of care	The services provided are in line with the health requirements and personal circumstances	The services provided does not conform to the health requirements and personal circumstances
Familiarity with doctor	Known doctor	Unknown doctor

Which provider profile do you prefer?

Example of a choice task presented to the respondents of the discrete-choice experiment

The questionnaire was developed in English and translated into two most used local languages (Hindi and Gujarati). The questionnaire was then piloted with 15 target group respondents to see whether they could understand the questionnaire and to check for their answer validity. Pre-test showed that all respondents understood the task and provided valid answers, but it also led to changing the wording of several attributes to eliminate any ambiguity in meaning and enhance respondents’ comprehension before data collection. Table 4 shows the attributes and their levels in the questionnaire.

**Table 4:** Attributes and their levels in the questionnaire

Attributes	Base Profile	Alternative profiles						
		1	2	3	4	5	6	7
Provider cost and type	0	0	0	0	1	1*	1*	1
Distance to the facility	35	35	15	15	35	35	15	15
Attitude of doctor and staff towards the patient	0	0	1	1	1	1	0	0
Appropriateness of care	1	0	1	0	1	0	1	0
Familiarity with doctor	1	0	1	0	0	1	0	1

Note: for coding refer to Table 1

#### Sampling

**Fig. 1:**
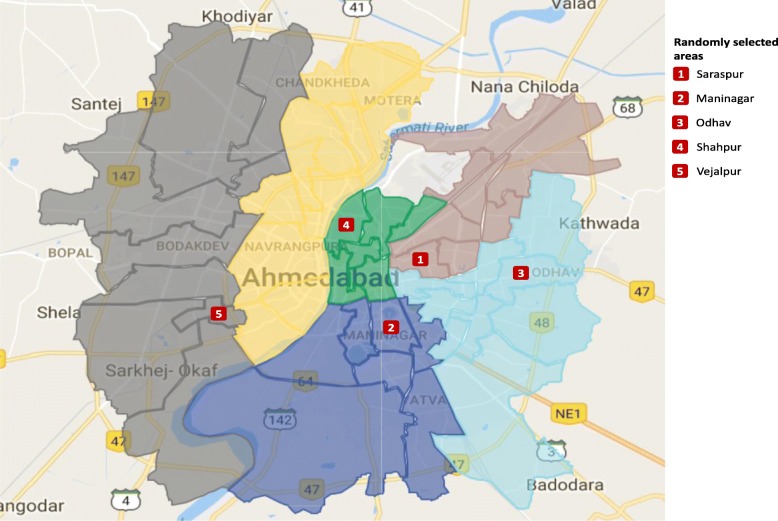
Ahmedabad city zone & ward map including randomly selected areas (Source: Amdav Municipal Corporation, 2017). Website: (https://ahmedabadcity.gov.in/portal/index.jsp)

The sample size was set to 100 respondents influenced by feasibility and available resources to conduct face-to-face interviews in the local setting. Multi-stage randomized sampling was designed for this study. The study setting, Ahmedabad, was divided into six administrative zones and each zone was further divided into a number of administrative wards. In the first step, five administrative zones were chosen using systemic random sampling. Then, one ward in each of the five chosen zones was selected by applying the method of standard systemic random sampling. The selected wards are depicted in Fig. 1. Once wards were sampled, one slum in each ward was randomly selected. In each slum interviewer randomly selected a house, followed by a systematic selection of every 4th household thereafter. Twenty households were interviewed in each slum. The number of refusals was minimal. In cases when household refused to participate, the nearest available household was chosen. Within each household, one respondent with age of 18 years or above and having involvement in the selection decision of the healthcare provider was chosen.

### Data collection

For data collection, a local research company was employed to conduct the administer survey questionnaire due to language skills and extensive experience in data collection in the local setting. Prior to the field work, the interviewers from the company participated in training provided by the main researcher. The training covered a review of the DCE method and review on the questionnaire followed by a Questions and Answers session. The questionnaire was available in three languages (English, Hindi, and Gujarati) and respondents were free to choose the language they were most comfortable with. Prior to the field work, ethical approval for the study was gained from the ethical committee of the Indian Institute of Management Ahmedabad. Interviewers began the interview with a short explanation of the study and asked each respondent for oral consent to participate in the study. No incentive was offered for participation in the study.

### Data analysis

The survey results were first scanned for completeness and data entry errors and then uploaded to Stata (Version 13). DCE data were then analysed using binary outcomes logistic regression with random effects. Each choice was treated as a single observation and the choice of the profile acted as a dependent variable (coded as ‘0’ when the base profile was chosen, and ‘1’ when alternative profiles is chosen). Independent variables in this model were the difference between the levels of each attribute in each choice set. Since the attribute of provider type and cost had three levels, two dummy variables indicating attribute differences had to be created for that attribute, while other attributes had one dummy variable indicating attribute difference. Besides the main-effect model, which only included attributed differences as independent variables, it was found that socioeconomic characteristics could influence the decision in accordance with previously reviewed studies, as explained earlier. Hence, the second model included interaction terms between the socioeconomic characteristics (expressed binary) and differences between the levels of each attribute used in the main-effect model. This second model was also parameterized using binary outcomes logistic regression with random effects. Finally, a reduced model was generated step-wise, by excluding statistically insignificant attribute differences and interactions. Specifically, variables were excluded one at a time in steps until only *P*-values lower or equal to 0.1 remained.

## Results

In total 100 complete responses were collected. However, seven responses were excluded from the analysis due to the lack of consistency between the first and last choice sets which were identical. This implied that these respondents did not perhaps understand the task. The descriptive statistics of socioeconomic characteristics of the 93 respondents included in the study are captured in Table 5.

**Table 5:** Descriptive information on socioeconomic characteristics of respondents included in the analysis (*N* = 93)

	n	%
Age (Mean/SD)	39.47 / 13.05	
Gender		
Male	55	59.14
Female	38	40.86
Marital Status		
Married	77	82.80
Divorced	1	1.08
Widowed	1	1.08
Single	14	15.05
Education level		
Illiterate	28	30.11
Can read and write	11	11.83
Primary (1–7 years)	23	24.73
Secondary (8–10 years)	22	23.66
Higher Secondary (11–12 years)	7	7.53
College	2	2.15
Household Income Monthly*		
Rs. 0–5000 (EUR 0–68)	62	66.67
Rs. 5000–10,000 (EUR 68–136)	27	29.03
Rs. 10,000–15,000 (EUR 136–204)	2	2.15
Rs. 15,000–25,000 (EUR 204–340)	2	2.15
Household size (Mean/SD)	5.70/2.53	

*Conversion on 28th June, 2017, 1 EUR = Rs. 73.49 (https://www.xe.com/currencyconverter/)

The average age of respondents was 39.5 years old, with an average household size of 5.7 people. The sample contained 59.1% males and 40.9% females. The majority of respondents were married (82.8%), while 17.2% were either divorced/widowed or single. Given that the target group was BoP populations, around 30% of respondents were illiterate and a total of 42% did not receive any formal education. Also, in accordance with the target group, 66.7% of respondents were in the lowest income group of Rs. 0–5000 monthly.

Table 6 shows the DCE results for the random effects logit model without interactions. Coefficients for all independent variables are statistically significant and of the anticipated direction (sign) except for provider type-and-cost attribute, which is not statistically significant.

**Table 6:** DCE results for the random effects logit model without interactions

Choice of profile (0 = base profile; 1 = alternative profile)	Regression Coefficient (95% CI)	Standard Error
Independent variables		
Δ PROVIDER_PRIVATE *Private instead of public provider*	−0.17 (− 0.62–0.58)	0.31
Δ PROVIDER_TRADITIONAL *Traditional instead of public provider*	0.11 (− 0.47–0.69)	0.30
Δ DISTANCE *20 min increase*	−0.07* (− 0.10 – − 0.04)	0.01
Δ ATTITUDE *Friendly instead of indifferent attitude*	1.81* (1.23–2.40)	0.30
Δ APPROPRIATENESS *Services in line with health requirements instead of not conforming with health requirements*	3.42* (2.67–4.18)	0.38
Δ FAMILIARITY *Known doctor instead of unknown doctor*	2.85* (2.11–3.59)	0.38
Observations (respondents)	651 (93)	
Log-likelihood function	− 221.03	
Wald Chi^2^	136.34	
Prob > Chi^2^	0.00	
Rho	0.07	0.07

*p < 0.05

The attribute which is relatively valued the most in the choice of provider, is the appropriateness of care (β=3.42, *p* = 0.00), followed by familiarity with doctor (β=2.85, *p* = 0.00) and attitude of doctor and staff towards the patient (β=1.81, *p* = 0.00). Negative coefficient for distance to the facility (β= − 0.07, *p* = 0.00) suggest that respondents prefer a shorter distance. The relatively low magnitude of the coefficient for distance to the facility, however, indicates that respondents are willing to travel longer if any of the other statistically significant attributes are present. It can also be mentioned that all statistically significant attributes are underlying determinants behind the choice of provider.

Table 7 shows the DCE results for the random effects logit model with interactions. It is important to mention that after accounting for interactions with socioeconomic variables, the statistically significant attributes in the previous regression model (see Table 6), do not show significance at a sample level but only within given socioeconomic groups. At the same time, the provider type-and-cost attribute, which is statistically insignificant in the previous regression model, emerges here as statistically significant at a sample level indicating preferences for a public provider. However, socioeconomic differences in preferences for that attribute are also observed.

**Table 7:** DCE results for the base model with interactions

Choice of profile (0 = base profile; 1 = alternative profile)	Regression Coefficient (95% CI)	Standard Error
Independent variables		
Δ PROVIDER_PRIVATE	−0.76* (−1.45 – −0.06)	0.36
Δ PROVIDER_TRADITIONAL	−2.07* (−3.19 – −0.94)	0.58
Δ PROVIDER_TRADITIONAL * dummy older	1.18* (0.13–2.23)	0.54
Δ DISTANCE * dummy older	−0.04** (− 0.09–0.00)	0.02
Δ ATTITUDE * dummy older	1.39* (0.50–2.27)	0.45
Δ APPROPRIATENESS * dummy older	2.79* (1.75–3.84)	0.53
Δ FAMILIARITY * dummy older	1.42* (0.46–2.37)	0.49
Δ PROVIDER_PRIVATE * dummy female	1.19* (0.06–2.31)	0.58
Δ PROVIDER_TRADITIONAL * dummy female	1.07** (−0.06–2.20)	0.58
Δ DISTANCE * dummy female	−0.05** (− 0.11–0.01)	0.03
Δ ATTITUDE * dummy female	1.17** (− 0.02–2.35)	0.60
Δ APPROPRIATENESS * dummy female	1.13** (− 0.20–2.47)	0.68
Δ FAMILIARITY * dummy female	2.46* (1.12–3.80)	0.68
Δ PROVIDER_PRIVATE * dummy not married	1.06* (0.05–2.07)	0.52
Δ PROVIDER_PRIVATE * dummy formal education	1.40* (0.38–2.42)	0.52
Δ PROVIDER_TRADITIONAL * dummy formal education	−0.08* (− 0.13 – − 0.02)	0.03
Δ DISTANCE * dummy formal education	1.99* (0.90–3.08)	0.56
Δ ATTITUDE * dummy formal education	3.31* (2.09–4.54)	0.62
Δ APPROPRIATENESS * dummy formal education	2.43* (1.26–3.60)	0.60
Observations (respondents)	651 (93)	
Log-likelihood function	− 219.70	
Wald Chi^2^	128.07	
Prob (Chi^2^)	0.00	
Rho	0.06	0.08

*p < 0.05; **p < 0.1

Specifically, older respondents have less strong preferences for a public provider over a traditional provider compared to younger people aged 18–39. The same can be said about female respondents compared to male respondents. However, female respondents also show preferences for private providers over public providers compared to male respondents. The same preferences for a private provider over public provider are observed for not married people compared to married people, as well as for those with formal education compared to those without formal education. On the other hand, respondents with formal education show a slightly stronger preference for public providers over traditional provider than those without formal education.

When it comes to the attribute of distance to the facility, it can be seen in Table 7 that respondents aged 40–65 prefer shorter distance more than younger people. A similar level of coefficient is found in the distance interaction with gender, showing that female subjects are more sensitive to the distance to the facility as compared to male. Moreover, shorter distance to the provider is more important factor for respondents without formal education than people with formal education.

Older respondents and females have somewhat stronger preference for friendly attitude towards the patient and services which are in line with health requirements and personal circumstances. Similarly, formally educated subjects have a stronger preference for friendly attitude of doctor and staff and for appropriate care than people without formal education. In addition, the attribute of familiarity with doctor in interactions with socioeconomic characteristics show that female as well as older respondents prefer known doctor more than male or younger people.

## Discussion

The results of this study indicate the stated preferences of BoP population living in urban slums in India for the service characteristics determining the choice of provider. This study is the first to quantify the relative utility of relevant service characteristics and to present results that provide new insights on how BoP consumers in India value attributes associated with a health care provider. The findings of the current study advance the current understanding of provider choice in urban slums in developing countries in several ways.

First, no homogeneous preferences emerged for the type of provider as some socioeconomic groups (female, not married and those with formal education) showed preferences for private providers, while other groups demonstrated preferences for public providers. Nevertheless, service provision by a traditional provider was not well accepted by the sample. These results are contrary to the findings in neighbouring Nepal, where a study revealed that traditional medicine prevail as treatment option in both peri-urban and rural areas [11]. In addition, socioeconomic characteristics prove to be significant in predicting provider choice and they should be carefully considered in understanding the patient choice behaviour, and the heterogeneity of the patient base.

Second, the statistical significance of the attributes used in the study indicates that they are indeed important and people do rely on them to base their choice. Thus, the results may shed light on why the majority of India’s population is reluctant to visit public hospital [1, 3], and why BoP population do not necessarily prefer traditional medicine, as anticipated based on earlier studies. A study from the urban poor in four cities in India revealed similar results that private facilities are being used extensively due to lack of government facilities nearby and the perception that private providers are able to offer more appropriate care [41].

While distance to the facility, attitude of doctor and staff towards a patient, appropriateness of care and familiarity with doctor were all found to be significant determinants behind the choice of health care provider, BoP consumers valued appropriateness of care above other determinants, followed closely by familiarity with doctor. Similar attributes were found to be important in previous studies. Appropriateness of care can be considered as representing technical quality of care, which stands for doctor’s skills, knowledge and appropriate use of care and medicines [28]. In line with this research, a preference study that took place in rural Liberia revealed a strong preference for technical quality of care, in the form of rigorous physical examination and prescription of drugs. Even though it is thought that patients are not able to judge the quality of these attributes, the study found that patients value the efforts made by provider [28]. Similarly, a study conducted in Zambia showed that technical quality of care conveyed through thorough examination was valued above other attributes such as attitude of doctor or cleanliness of the facility [27]. Furthermore, a study in South Africa found that drug availability is the main concern for local populations due to lack of available medication in a real-life situation, followed by the thorough examination [42]. These prior studies and the results of our study indicate that appropriateness of care and technical quality of care is a concern in low-income settings.

Familiarity with doctor has also been found as an important attribute underlying the choice of provider. During data collection phase, it was observed that respondents often would make a comment that in most cases they would choose a familiar doctor known to them. This was also found in qualitative study conducted in the same region which revealed that recommendations by neighbours or family play an important role in choosing the health care provider [39]. This attribute could also be linked with continuity of care. It could be assumed that visiting a familiar doctor not only creates trust in the relationship between doctor and patient, but also contribute to the continuity of care and patient centeredness. Preference for familiar doctor was found in several previous studies. For example, DCE study results from rural Ethiopia showed the preference for good physician and nursing communication as well as for full drug availability and continuity of care [29]. In another instance, a study on the choice and preference for public-private health care among urban residents in China also showed that respondents showed significant preference for knowing a doctor [25]. Making sure that BoP consumers stick to the same General Practitioner (GP) over the longer periods of time would not only satisfy their preference for familiar doctor, but could also contribute to continuity of care which is commonly linked to higher patient satisfaction [43]. BoP consumers give more importance to the relationship with the healthcare provider.

Respondents in this study also significantly valued attitude of doctor and staff towards the patient. Through the interactions model it was also found that female have stronger preference for friendly behaviour of doctors and staff compared to men. These findings are in line with some previous studies. For instance, respectful treatment by clinic staff was identified as a significant attribute in the study in Liberia [28]. In two other instances, studies resulted in provider attitude being the most important driver valued even above technical quality attributes [44, 45]. However, both of these studies investigated preferences of women in Tanzania in their choice of provider for delivery care. Friendly and respectful staff attitude was also identified as essential characteristic of provider in both South Africa [42] and Zambia [27].

### Limitations and directions for future research

Like other studies, there are several limitations of this study that need to be acknowledged. The DCE questionnaire only included five attributes and defined limited attribute levels which means that the study results should be interpreted in relative terms, i.e. in light of the attributes included in the study [46]. Therefore, to mitigate the risk of missing essential attributes, we carried out a thorough process of selecting attributes relevant to the study setting. While we based the choice of attributes on the opinion of healthcare consumers and academic researchers doing research in healthcare domain, the opinion of policymakers or local institutions has not been explicitly taken into account. Another limitation is that a no-choice option was not included in the questionnaire. Hence, respondents who would choose not to seek care were forced to make a choice leading to results that might not explain some of the subject’s behaviour. Thus, our results should be viewed in terms of hypothetical choices as opposed to real-life situations [29]. This bias was partly limited by informing respondents on the goal of the study prior to filling in the questionnaire. We also made efforts in choosing attributes and attribute levels which matched the real-life situation in the local setting. Furthermore, we also recognize that the results are only relevant to the researched region and may not necessarily be generalizable to populations in other Indian regions, especially rural setting. However, as discussed above, the results are in line with similar studies, which supports their convergent validity.

## Conclusion

This study administered a DCE to examine the factors affecting the choice of health care provider in a low-income setting, specifically the urban slums in India. The respondents who were representatives of BoP populations valued appropriateness of care above other attributes. This was followed by the familiarity with doctor and attitude of the doctor and staff towards the patient. As expected, respondents prefer shorter distance but were willing to travel longer if any of the other preferred attributes were present. The study did not reveal universal preferences for a provider type, but overall the traditional provider type appeared not well accepted. Thus, the attempts of the Indian government to emphasize the focus on traditional providers such as Ayurveda, Naturopathy, etc. should be carefully reviewed as the study did not reveal a clear preference for such services. This may also be due to limitations of traditional healthcare system which often are useful in the treatment of relatively less complex health care problems and less effective to provide faster relieve, especially in the acute care problems.

Policy makers need to work on aligning the capabilities of public health system with expectations of BoP populations. The results of our study and other similar studies could be used by policy-makers to better understand the provider choice and prioritize improvements of health care service characteristics. Specifically, future research should include additional service attributes in the DCE design such as waiting time or status of medical equipment. Moreover, further research should investigate population preferences in BoP setting when faced with acute or emergency conditions. It is important to understand whether preferences found in this study are unique to BoP consumers and how these preferences would be different for non-BoP consumers in low-income countries. This will indicate the need for targeting the BoP consumers through appropriate policies.

## Abbreviations

AYUSH: Ayurveda, Yoga, Unani, Siddha, and Homeopathy
BoP: Bottom of Pyramid
DCE: Discrete-choice Experiment
